# WriteSim TCExam - An open source text simulation environment for training novice researchers in scientific writing

**DOI:** 10.1186/1472-6920-10-39

**Published:** 2010-05-28

**Authors:** Jatin Shah, Dimple Rajgor, Meenakshi Vaghasia, Amruta Phadtare, Shreyasee Pradhan, Elias Carvalho, Ricardo Pietrobon

**Affiliations:** 1 Department of Research, Duke-NUS Graduate Medical School, (8-College Road), Singapore, (169857), Singapore; 2 Kalpavriksha Healthcare and Research, Thane, (421202), India; 3 Research on Research Group, Duke University Medical Center, Durham, NC, (27710), USA; 4 Department of Surgery, Duke University Health System, (DUMC Box 3094), Durham, NC, (27710), USA

## Abstract

**Background:**

The ability to write clearly and effectively is of central importance to the scientific enterprise. Encouraged by the success of simulation environments in other biomedical sciences, we developed WriteSim TCExam, an open-source, Web-based, textual simulation environment for teaching effective writing techniques to novice researchers. We shortlisted and modified an existing open source application - TCExam to serve as a textual simulation environment. After testing usability internally in our team, we conducted formal field usability studies with novice researchers. These were followed by formal surveys with researchers fitting the role of administrators and users (novice researchers)

**Results:**

The development process was guided by feedback from usability tests within our research team. Online surveys and formal studies, involving members of the Research on Research group and selected novice researchers, show that the application is user-friendly. Additionally it has been used to train 25 novice researchers in scientific writing to date and has generated encouraging results.

**Conclusion:**

WriteSim TCExam is the first Web-based, open-source textual simulation environment designed to complement traditional scientific writing instruction. While initial reviews by students and educators have been positive, a formal study is needed to measure its benefits in comparison to standard instructional methods.

## Background

Biomedical researchers need strong writing skills to obtain funding and to communicate the results of their research [1-3]. The success of grant proposals and research manuscripts depends as much on the quality of the writing as on the promise of the research or the significance of the results. Yet there is a lack of relevant extensive [4] and effective teaching mechanisms [5] in the area of scientific writing. When formal instruction is available, it is often reductive and mechanistic, [6] and fails to impart basic knowledge of rhetorical techniques, structure-content differentiation, style, clarity, and accuracy [7]. Researchers need a firm grounding in these concepts to participate in highly specialized scientific communities [8]. There is a clear need for instructional methodologies that incorporate hands-on experience, familiarization with existing literature, consistent practice, topical relevance, and explicit learning methods [9-12].

Innovations in information technology have given rise to new tools for science education. Increasingly sophisticated simulation environments, for example, have been used in a variety of disciplines, including engineering [13] economics, [14] and physics e.g., electrical circuits [15]. Flight simulators are used to train pilots and astronauts; [16-18] war games train military personnel; [19] and management games train business managers and decision makers [20,21]. More recently, simulations have been used in clinical settings, such as critical care [22] and emergency medicine, [23] to train residents and medical students, and they have proven effective in teaching nurses how to respond to uncommon, composite clinical situations[24].

Simulation environments are designed to mimic real-world systems with sufficient accuracy to provide the rough equivalence of hands-on experience [25,26]. They try to duplicate problems trainees will encounter in the real world, which improves their problem-solving skills; [27] as well, they can be programmed to simulate any situation, they standardize training routines, and they can be accessed at any time convenient to the user. Used consistently, they can substantially develop skills. Current practices in teaching scientific writing are largely based on trial and error, which discourages young researchers and makes poor use of their time. There are currently no simulation environments for developing writing skills. Text structure templates that serve to guide researchers on the role of a text block in a manuscript have been previously developed and tested by the research on research group (RoR) [28,29]. However during informal use of these templates while coaching researchers, our group noticed that researchers appreciated the help of templates but at times found it difficult to populate the text blocks with relevant content. This stimulated our interest in exploring the role of simulation environments in coaching researchers in manuscript writing.

Given their success in other biomedical sciences, and their proven record of honing necessary skills, we believe simulation environments can be a powerful tool for scientific writing instruction. This paper presents our contribution in the form of WriteSim TCExam, an open source, web-based, textual simulation environment

## Implementation

### Design objectives

We set out to develop a simulation environment to educate novice researchers as to appreciate and use the proper structure, content, and style of scientific writing. We aimed to design a simulation environment that is: 1) web-based; 2) open-source; 3) well-structured and documented; 4) user-friendly; and 5) amenable to a question-and-answer format. Additionally, rather than starting from scratch, we decided to modify an existing application. We reviewed GoVenture simulation designer, [30] a commercial desktop-based application with which nontechnical users can build custom learning simulations. We also reviewed TCExam, [31,32] a Web-based application that enables educators to create, schedule, deliver, and produce reports on surveys, quizzes, and exams. Finally we chose TCExam for its simple, intuitive interface and its open-source architecture.

We reviewed existing applications to find examples of features that could be added to TCExam to enable it to function as a textual simulation tool. We assessed the feasibility of each feature especially considering the scope of our objectives as well as the time and funds available. In the end, we decided to preserve the basic architecture and interface of TCExam [31], with the following modifications:

1. End users would receive immediate feedback upon answering questions. Incorrect answers would produce detailed explanations, and the user would then select from the remaining answers.

2. A grading mechanism would provide a summary of the user's performance, identifying areas for further improvement.

3. Blogs and forums would enable mentoring relationships via interactions among participants and between participants and the administrator. This would facilitate the exchange of ideas and help answer participants' questions in a friendly environment.

4. Persistent bugs in the existing version of TCExam would be corrected, and the user interface would be modified to make it more user-friendly.

After incorporating the above changes, we renamed the application as "Writesim TCExam." We maintained a list of potential further modifications, to be effected later, that were excessively time consuming or expensive, or beyond the current scope of the project.

### Characteristics of Writesim TCExam users

Writesim TCexam has two distinct interfaces: admin interface and user interface, each have different set of uses. The 'admin interface' is meant to be used by senior researchers, mentors or course instructors (from now on referred to as "administrators") involved in training novice researchers in scientific writing. They can design the simulation material, upload it, design the simulation test and share it with novice researchers (from now on referred to as "users") who can access it through the 'user interface.' The 'user interface' helps the users to access and interact with the simulation material developed by the administrators.

### Steps to design and implement a textual simulation environment

#### Step 1: Design the simulation material

Writesim TCExam allows administrators to easily design and manage simulation material related to manuscript and grant writing. For the purpose of Writesim TCexam, we define simulation material as examples designed from previous publications using the question - answer - answer key format.

By examining a series of examples, novice researchers can 1. learn to distinguish between good and poor scientific writing and understand the placement and flow of content in a manuscript. It equips them with the tools and perspective to evaluate and improve their own writing.

In parallel to modifying TCexam, we developed simulation material from 30 randomly selected peer reviewed publications published in Biomed central [33] and indexed in PubMed [34]. Next we analyzed the structure of each manuscript and dissected it to determine the text blocks [28] that drive the argument flow. We used these text blocks to formulate 100 simulation questions. These questions were focused on helping users to understand the role of various text blocks in a manuscript and how to populate the text blocks with relevant content.

We then prepared a list of possible answers to the questions. Some of these answers were very close to the right answer while others were distinctly unrelated. The underlying purpose was to enable users to look at a wide range of common errors and understand the distinction between ideal and not so ideal scientific writing. We also designed answer keys that would be displayed in a pop-up window, when the users selected wrong answers. We believe, the answer keys would enable the user to learn and understand the underlying reason behind correct/incorrect answers which would facilitate its application in their subsequent efforts at scientific writing. Next we classified the material into topics corresponding to the IMRD (Introduction, Methods, Results and Discussion) structure and uploaded them to Writesim TCExam. Since, initially our purpose was to test the modified TCExam application, we randomly selected and retrieved publications from pubmed. However, in future practice, we propose the use of high impact peer reviewed publications for the development of simulation material. We believe that it can enable users to appreciate the architecture of high impact publications while learning the nuances of scientific writing.

#### Step 2: Add the simulation material through Admin interface

Administrators can log in to the admin interface which has 2 major sections - a central work area and a navigation pane on the left. The navigation pane aids navigation through six major sections of the application: index, users, topics, tests, help, and info. The Index section, (displayed by default) provides a brief overview of the six sections and their respective subsections in the work area. Selection of a specific section/subsection displays a brief description of its purpose and functionality at the bottom of the screen. Detailed information about a majority of features and functionalities can be accessed from the documentation on the TCExam website [32].

Following sub-steps describe how simulation material can be added to Writesim TCexam:

##### i. Define topics

**Figure 1:**
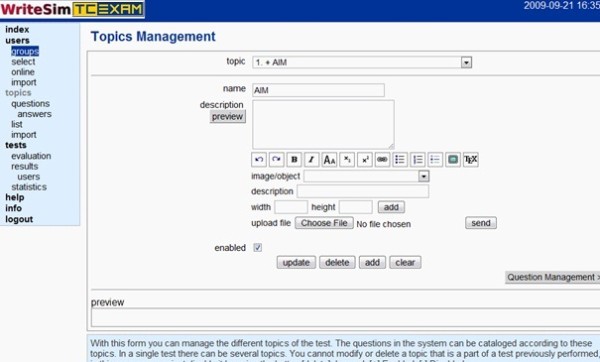
**Writesim TCExam - Workflow diagram for users**.

Writesim TCexam follows a topic-question-answer-answer key hierarchy. After defining the name of the topic, administrators can add a brief description and an image if needed. (Figure 1)

##### ii. Add questions

**Figure 2:**
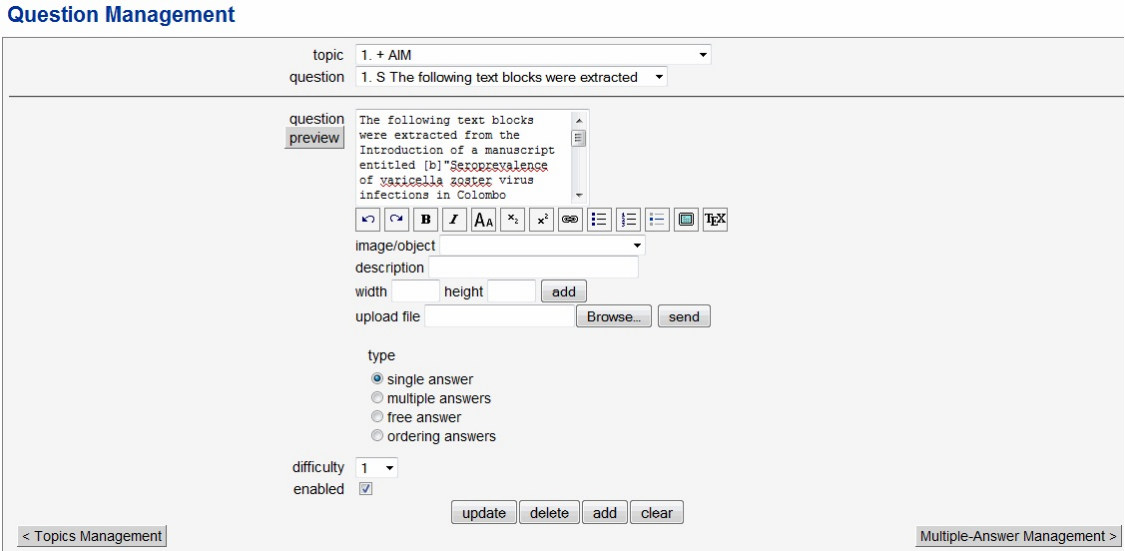
**Users section of TCExam**.

After defining the topics for the simulation material, questions related to each topic can be uploaded in the question management ('questions') section. This section can be accessed from an icon just below the topics page or from the navigation pane. Administrators can add the question description along with images if any. Answer types, difficulty levels and disable/enable are additional features. Answer types range from single answer, multiple answer, free answer and ordered answers. (Figure 2)

##### iii. Add answers and answer keys

**Figure 3:**
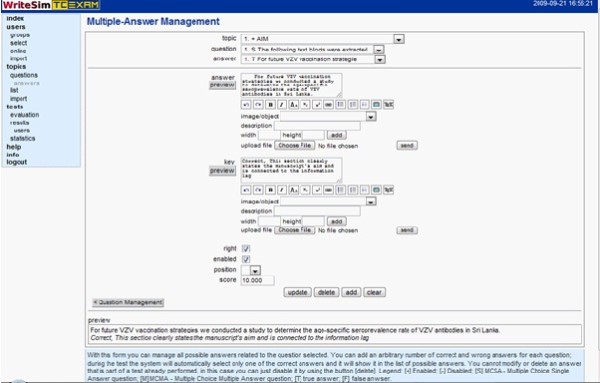
**Adding answers and answer keys in Writesim TCexam**.

In case of multiple answer type questions, administrators can add them along with their keys in the multiple answer management form. ('answers'). Administrators can also upload images, define right/wrong answers, score answers, define their position in the list of answers and enable/disable answers. Administrators can review all the uploaded material through the 'list' section in the navigation pane. (Figure 3)

#### Step 3: Implement simulation test

After uploading the simulation material, administrators can implement the simulation test for a single user or amongst a set of users ('groups'). For this purpose, following sub steps can be followed:

##### i. Add users and groups

**Figure 4:**
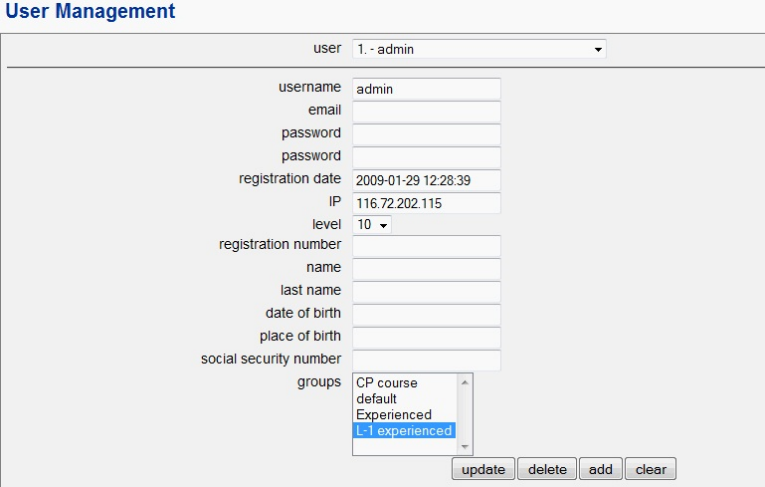
**Adding users and groups for a simulation test in Writesim TCexam**.

Administrators can provide access to users/groups of users by filling up the 'User' form. A list of users can also be imported into Writesim TCexam. (Figure 4)

##### ii. Design a simulation test

**Figure 5:**
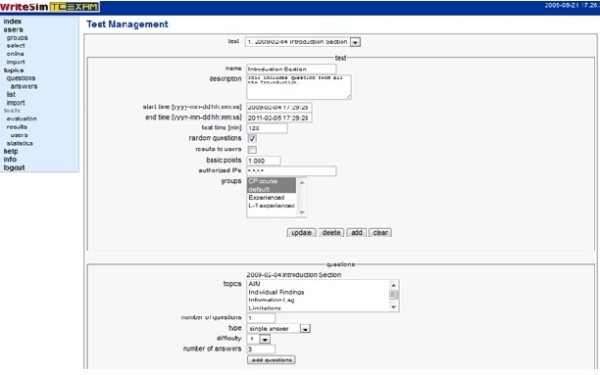
**Designing a simulation test in Writesim TCexam**.

Administrators can use the test management form to design a simulation test. Test name, a brief description of the simulation test, period of user access and total test time are some of the fields of the test management form. Administrators can choose to randomly select simulation questions from the list of simulation questions previously designed. They can also define the score points for each question and choose whether results should be displayed to users at the end of the test. Access for the simulation test can be provided to specific user groups. Finally administrators can choose to include specific topics from a list of topics, number and type of questions and the difficulty level of questions. After designing the test, administrators can send users a link to the user interface along with their log in details [35] along with the log in details. (Figure 5)

### Steps to use the textual simulation environment

**Figure 6:**
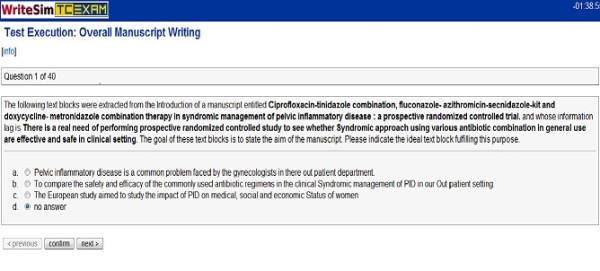
**User interface of Writesim TCExam**.

**Figure 7:**
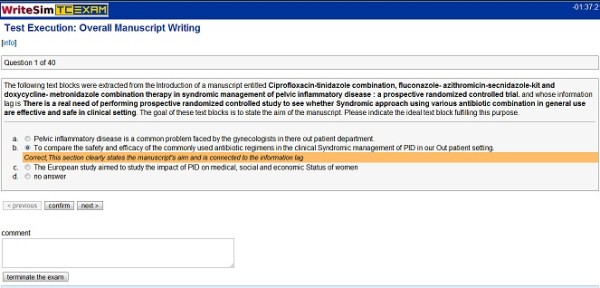
**User interface with Key**.

Users can access Writesim TCexam by following the luser interface link provided by administrators. The simulation test displays a list of questions that they can access one after the other. After selecting a specific question from the list, a description of the question along with possible answer options are displayed. Users can choose the answer option that they think best answers the question. In case if the answer is wrong, an answer key justifying the same is displayed. Users are given an additional chance to choose the right answer; failure at which displays the correct answer key. Users have the choice to answer a question or skip the same. They can also leave comments related to a specific question. Finally they have the choice to answer all questions in the list or end the test midway and submit it. lets add.. The following images display the user interface (Figure 6) and the user interface with key (Figure 7), of the Writesim TCExam respectively.

### Evaluation of simulation test completed by the users

While designing the simulation material administrators can grade the questions and accordingly generate scores at the end of the simulation test. Once a simulation test is successfully conducted, administrators can access individual results for each user and view the test score. They can send the results to the user as a PDF file through an email.

### How Writesim TCexam works as a simulation environment?

TCExam's question-and-answer format based on computer aided formative assessment method is a promising foundation upon which to build a scientific writing simulation environment. Formative assessment aims to improve learning rather than grading it and is intimately related to instruction. Well conceived and designed question and answer fields can be used to present examples of text blocks from different sections of a manuscript, and instantaneous feedback clarifies concepts while providing positive reinforcement [36,37]. The difficulty that users of Writesim TCExam experience in distinguishing between ideal and less-than-ideal text structures as well as content placement simulate the challenges faced by novice scientific writers when they write their first manuscripts. The power of reinforcement through immediate feedback has often been exploited by behavioral scientists in the design of computer-based instruction tools, [38-41] and early process-based studies have also demonstrated the power of instant and corrective feedback [41,42].

The success of this application depends on well conceived and well designed simulation material which can help students to understand various aspects of scientific writing. For example: the role of a manuscript's various sections (see templates [43] for a description); the proper framing of subsections and content placement. The list of topics can be extended as per the scope and goal of scientific writing instruction. To develop this material, our group drew on its expertise in standardized training methods (such as manuscript dissection and templates) that help novice writers to structure and organize their thoughts

### Blog and forum

Writesim TCExam's blog and forum can be accessed from the admin and user interfaces. The blog is based on the wordpress software script, [44] and the forum is based on PHP Bulletin Board, [45] an open-source forum package. The blog serves as a platform for administrators to post guidance material, videos, slides on scientific writing. The forum serves as a platform for users to communicate their experiences, difficulties or questions about the simulation test or scientific writing in general. Administrators can provide comments or address difficulties at an individual level in these forums. Users can provide inputs and feedback on the simulation questions and test which can help administrators in improving simulation material.

### Proposed workflow

#### Administrators

**Figure 8:**
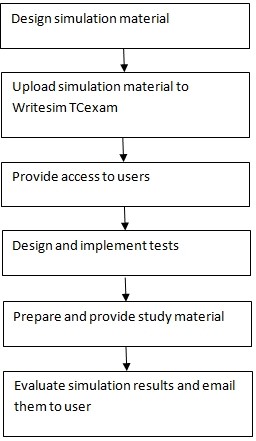
**Admin user workflow**.

After designing the simulation material, administrators can log in and upload it to relevant sections of Writesim TCExam through the admin interface. They can then 1. design simulation tests 2. define users and 3. implement the test by providing access to the users. Administrators can complement existing courses/educational-training programs in scientific writing by providing access to a textual simulation environment like Writesim TCExam customized to their needs. In preparation to the simulation test, aministrators can provide users with study material on scientific writing which may consist of 1. list of peer reviewed articles focused on scientific writing, for example "The science of scientific writing"[46] 2. slides, videos prepared by administrators that explain scientific writing 3. difference on structure - content and scientific writing templates [28] and 4. recommended books on scientific writing. Administrators can also provide instructions on how to use the application through an inclass session, slides or videos. Once the users have successfully used the simulation material, administrators can evaluate their test results to score/grade them. Subsequently administrators can choose to email the results to users. (Figure 8)

#### Users

**Figure 9:**
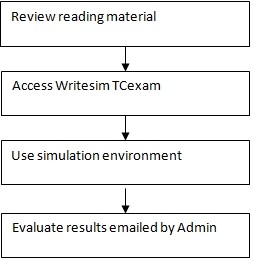
**Simulation user workflow**.

Before using the simulation environment, users are expected to review reading material or such other guidance material on scientific writing shared with them. Subsequently they can use the simulation environment based on the link and access provided by the administrators. After using the simulation environment, they may receive the results for self evaluation either immediately or later by email. (Figure 9)

#### Field usability

After the application was created, the first three months of development were devoted to conducting field usability tests which revealed major/minor problems with software coding, uploading images, logging in, and navigation. Once these problems were corrected and the navigation interface optimized, we conducted tests using example simulation material contributed by senior researchers in the (RoR) group [47]. These tests revealed additional issues concerning use of special characters, navigation, and display errors which were identified and corrected.

#### Usability

To date, Writesim TCExam has been used informally to train 15 novice researchers from our research group (RoR). Simulated topics include topics like: role and framework of manuscript subsections as well as content placement in scientific manuscripts. Trainees report that the application was easy to use, it helped them understand structure and content, and it improved their overall writing skills.

#### User survey

##### Admin

Admin testing was performed by 15 junior and senior researchers who matched the 'administrator' profile described earlier and who met minimum standards of computer literacy. They completed an online survey (Additional file 1) using DADOS-Survey, a CHERRIES-compliant survey tool [48]. They were shown a 15-minute tutorial [49] and instructed to explore the application. They described their experiences as summarized in Table 1.

**Table 1:** Admin survey results

Survey Questions	Responses: Scores (percentages)				
	SD	D	N	A	SA
The speed of the application is excellent (N = 15)	0(0.0)	0(0.0)	1(6.6)	8(53.3)	4(26.6)
WriteSim is extremely easy to learn	0(0.0)	2(13.3)	0(0.0)	9(60.0)	2(13.3)
WriteSim is extremely easy to use	0(0.0)	0(0.0)	1(6.6)	9(60.0)	3(20.0)
It is very easy to understand all functionality available within Writesim	0(0.0)	1(6.6)	2(13.3)	7(46.6)	2(13.3)
The navigation in WriteSim is highly intuitive	0(0.0)	0(0.0)	0(0.0)	12(80.0)	1(6.6)

SD = .Strongly Disagree, D = Disagree, N = Neutral, A = Agree, SA = Strongly Agree

##### Users

User testing was performed by 14 novice researchers who matched the profile of 'users'. They completed an online survey (Additional file 2) using DADOS-Survey, volunteering biographical information and their assessment of the user interface. They described their experiences as summarized in Table 2.

**Table 2:** User survey results

Survey Questions	Responses: Scores (percentages)				
	SD	D	N	A	SA
The speed of the application is excellent (N = 10)	0(0.0)	0(0.0)	0(0.0)	4(40.0)	6(60.0)
WriteSim is extremely easy to learn	0(0.0)	0(0.0)	0(0.0)	9(90.0)	1(10.0)
WriteSim is extremely easy to use	0(0.0)	0(0.0)	0(0.0)	6(60.0)	4(40.0)
Based on your brief interaction with the simulation material, do you think it helped you better understand the role of various subsections of a scientific manuscript?	0(0.0)	0(0.0)	1(10.0)	7(70.0)	2(20.0)
Based on your brief interactionwith the simulation environment, do youthink it helped you better understand how specific scientificcontent fits into different subsections of a manuscript?	0(0.0)	0(0.0)	2(20.0)	7(70.0)	1(10.0)
Do you think the keys (feedback mechanism) in the simulation tests are highly beneficial?	0(0.0)	0(0.0)	3(30.0)	2(20.0)	5(50.0)
The simulation material in Writesim is useful for learning scientific manuscript writing	0(0.0)	0(0.0)	0(0.0)	8(80.0)	2(20.0)
Based on your brief interaction with the simulation environment, would you look forward to using this application in the future?	**Yes:**9(90.0)	**No:**1(10.0)			

## Results

### Early usage

Writesim TCExam currently has simulation material designed by our research group to enable novice researchers to understand the role of various text blocks in a manuscript and content placement. The application currently has 100 questions derived from 30 open access articles published in the BMC journal. The application with these 100 questions has been used by us to train 30 novice researchers since 2007. These researchers hailed from various backgrounds like medical students, clinicians and residents. Based on informal communication, we understand that they benefited from using the simulation environment.

### Admin user survey

This survey was completed by 13 of the 15 participants(86.66%). Majority of the participants reported the application to be fast (agree = 53.33%, strongly agree = 26.66%), easy to learn (agree = 60%, strongly agree = 13%), easy to use (agree = 60%, strongly agree = 20%), easy to understand in every aspect of its functionality (agree = 46.66%, strongly agree = 13.33%), and easy to navigate (agree = 80%, strongly agree = 6.66%). Responses to questions regarding computer literacy suggest that most respondents were well-versed in basic computer functions. (Table 1)

### User survey

This survey was completed by 10 of the 14 participants(71.42%). Majority of participants were females (0.6%) and had post graduate degrees (0.7%). In terms of past publication most of them (0.8%) had never published a peer reviewed manuscript while some of them (0.2%) had published between 1-5 peer reviewed manuscripts. (Table 3)

**Table 3:** Demographic characteristics of User survey

Total users	n = 10	
Gender	females	6 (0.6%)
	Males	4(0.4%)
		
Education	Undergraduate	0
	Graduate	3(0.3%)
	Post graduate	7(0.7%)
		
Past peer reviewed publications	Never published	8(0.8%)
	Published 1 scientific manuscript	0
	Published more than 1 but less than 5	2(0.2%)
	Published more than 1 but less than 5	0

A majority of them reported the application to be fast (agree = 40%, strongly agree = 60%), intuitive to navigate (agree = 90%, strongly agree = 10%), and easy to use (agree = 60%, strongly agree = 40%). In addition, most felt that training with Writesim had improved their understanding of the functions of a manuscript's subsections (agree = 70%, strongly agree = 20%); that it improved their understanding of how content is divided into subsections (agree = 70%, strongly agree = 10%); that the answer keys that provide feedback were highly beneficial (agree = 20%, strongly agree = 50%); the overall experience helped them better understand scientific manuscript writing (agree = 80%, strongly agree = 20%); and they would look forward to using Writesim TCExam in the future (Yes = 90%, No = 10%). Responses to questions regarding computer literacy suggest that most respondents were well-equipped with basic computer skills.

## Discussion

### Summary

To create Writesim TCExam, we started with TCExam, an existing open-source, Web-based assessment application and modified it to function as a textual simulation environment. TCExam is used by educators to design, schedule, execute, and report assessment tests. Our decision not to use Goventure stemmed from the fact that it is a commercial, non-open source application. Commercial applications have a cost that limits distribution and use. Additionally since it was not open source, we couldnt make the modifications outlined earlier on our own. We named our adaptation as "Writesim TCExam" to denote its status as a writing simulation environment. After correcting the minor problems in the software, the user survey studies show the application to be intuitive, easy to navigate and use.

### Other instructional methods

The research community functions as a collective network, exploring, validating, and disseminating scientific ideas that benefit society. Effective scientific writing is fundamental to the progress of the scientific community and to the careers of individual scientists; [1-3] therefore it is essential that novice researchers develop their writing skills. As an added benefit, an in-depth understanding of the writing process can increase productivity [50].

Over the years, a great many methods for teaching writing skills have been explored, including traditional classroom instruction, seminars, workshops, certificate courses, distance learning, and mentoring. One method, collaborative learning, stresses collective problem-solving [51]. While it has shown some promise in teaching writing skills to researchers, [52] its practical application is limited because scientific communication depends, in the end, on individual effort. Simulation environments can complement collaborative learning by helping researchers understand the flow of ideas in scientific manuscripts, and the difference between structure and content. As well, studies have noted that simulation environments often promote collaborative learning, which prepares students for peer criticism and group work [53].

An increasingly popular method, e-learning, makes use of the Internet [54] and other electronic resources, such as multimedia [55]. However, it often amounts to nothing more than the digitization and dissemination of previously existing educational materials, and so fails to fully take advantage of new technologies, while often perpetuating inefficient and ineffective lesson plans. One example is the e-learning tool created by Dagmar Malikova, consisting of 11 self-study modules, which although is well-designed but is not interactive [56]. Another innovative but limited use of digital technologies involves searching Internet biology forums for comprehensible examples of scientific writing and then using computerized retention strategies to produce "digital learning logs" to track common errors [57].

Other methods like group manuscript critiques, [58] rewriting published manuscripts, [12] manuscript editing, [59] and journal clubs and letter writing [60] can help build writing skills, but they are insufficient on their own and must be combined with other methods. Similarly traditional practice assignments have also been shown to be insufficient to help in improvement of writing skills [61,62]. Finally, studies evaluating the effectiveness of these and other approaches have yielded few important findings, and the findings are often contradictory [63].

The great variety of writing instruction programs attests to the diversity of settings and objectives that collectively serve to educate novice researchers. Whatever the training method or context, it is important to remember that writing is a dynamic, individualistic process, to which each student brings his own perspectives and concerns, [64] and that, where possible, training programs should be tailored to the specific needs of the various specialties [62].

### Simulation environments

In comparison to the methods described above, simulation environments provide a realistic environment in which users can explore simplified versions of both realistic and highly hypothetical situations [65].

Researchers evaluating simulation-based approaches to second-language writing instruction, with an explicit focus on genre and genre analysis, cite numerous benefits. Students become increasingly aware of discipline-specific features, they develop competence in discourse, and they become more precise in their use of language [66]. Simulation also helps students overcome motivational and attitudinal problems, especially those related to collaborative learning [53,67-69]. Other studies have shown that simulation environments increase opportunities for collaborative learning, which improves students' attitudes toward peer criticism and group work [53]. The many strengths of simulation environments speak to their great potential for scientific writing instruction.

We chose TCExam as it followed the computer aided formative assessment method. It suits well for a simulation environment as it also encourages reflective style of learning. It enables consistent delivery and immediate feedback. Recent applications also allow the use of images and videos making the application rich and interactive. Repeatability, flexibility of access, reliability and being student centred are some of its many advantages [70]. By improving student learning outcomes, it leads to positive attitude towards learning [71]. These benefits add on to those of simulation environments. On the other hand, development time, risks related to hardware, software and administrative aspects of the application and need for users to be computer literate are some of its disadvantages [72] which are equally applicable to simulation environments. In reference to feedback in assessment applications, immediate explanatory feedback on why an answer is incorrect is more beneficial to users as compared to no feedback and it leads to better performance [73]. Although not focused on assessing users, feedback plays an important role in simulation environments. It would facilitates better understanding and retention of the concepts and various aspects of scientific writing. Additionally a second chance mechanism to choose the correct answer was aimed at encouraging brainstorming and enhancing the learning process.

The blog and forum are primarily aimed at improving and enhancing user-user and user-administrator communication. Since Writesim TCExam is an online application, users may not be located in the same place thus restricting group and collaborative discussions. Blog and forum address this issue and serve as a platform for voicing their queries, finding solutions to queries and exchange of individual experiences.

We think Writesim TCExam would be more accessible to the research community owing to its open source nature as compared to other commercial simulation environments like Goventure [30]. Individuals involved in teaching and training novice researchers like mentors, course instructors, program coordinators can download Writesim TCExam [74] and install it at their institutes. They can modify the application if required as well as develop simulation material according to their needs. They can administer the textual simulation environment by providing a link to the application along with instructions on how to use it. The end users can follow the link to undertake the simulation test.

### Limitations

Currently Writesim TCExam follows a test-feedback, *i.e*. deterministic mechanism which does not support real time analyses and feedback on non deterministic aspects of scientific writing. Structure and semantic interconnections that assist the reader to map and understand the context of content constitute the deterministic aspects of writing. Structure persists across multiple articles while content changes according to the topic and hence the latter constitutes the non-deterministic aspect of writing. Gopen [46] argues that deterministic aspects (structure) of written communication provide clues to the reader enabling them to make important interpretative decisions about the content. Our application thus focuses on mimicing intricacies of the deterministic aspects of scientific writing. Additionally, its effectiveness highly depends on the quality of the simulation material.

### Current utilization

Writesim TCExam is currently used by the RoR group to train novice researchers and medical students in scientific writing. Writesim TCExam will be used in the Certificate Course in Outcomes Research, [75] an eight-month course of study that will soon be implemented at the Duke-NUS Graduate Medical School in Singapore. The course trains healthcare professionals in every step of research publication, from generating a dataset to submitting to a high-quality journal. Writesim TCExam will be used as a pre-class exercise to train participants in manuscript writing.

### Potential uses

WriteSim TCExam is an inexpensive instructional tool that has potential to significantly improve researchers' confidence and writing skills and reduce the time required to produce high-quality manuscripts

## Conclusion

Writesim TCExam is a first-of-its-kind, Web-based, open-source textual simulation environment designed to complement traditional scientific writing instruction. While initial reviews have been positive, a formal comparative study is needed to measure the benefit to writing quality and related outcomes when compared with standard instructional methods alone.

## Availability and requirements

**Project name:**Writesim TCExam

**Project home page**: http://www.ceso.duke.edu/tcexam

**Operating systems**: Linux and Windows

**Programming language**: PHP

**Other requirements**: Apache Server, MySQL or PostGreSQL, XHTML, CSS, Sendmail, PHPMailer, TCPDF library, Barcode Render Class for PHP using the GD graphics library, and LaTeX Rendering Class v0.8.

**License**: GNU General Public License v.2. This license ensures that the source code can be freely distributed, modified, and sold, as long as the source code is provided with every copy of the application. The source code is available at no charge.

**Restrictions to use by academics/non-academics**: New users must email the Research on Research group for a user name and password.

**Source code**: http://www.ceso.duke.edu/tcexam/tcexam.tar.gz

Software links

**1. Admin link**: http://www.ceso.duke.edu/tcexam/admin/code/index.php

**• user name**: reviewer

**• password**: reviewer

**• Description**: By using the above link, log in and password, you can get the admin rights. You can create users/groups, assign passwords, create tests, assign tests to specific groups, add topics, add questions, see the results of participants and many other admin functions.

**2. User link**: http://www.ceso.duke.edu/tcexam/public/code/index.php

**• user name**: reviewer

**• password**: reviewer

**• Description:**The users/participants can execute the test assigned to them by using this link, log in and password. For example, there is a test "Introduction Section" already created to give an idea of the functioning of this software.

## Abbreviations

XHTML: Extensible HyperText Markup Language; CSS: Cascading Style Sheets; LATEX: Lamport Tex; PDF: Portable Document Format; PHP: Hypertext Preprocessor.

## Competing interests

The authors declare that they have no competing interests.

## Authors' contributions

JS contributed to the development and testing of the application and wrote the manuscript. DR contributed to the testing of the application and reviewed the manuscript. MV contributed to the development and testing of the application and reviewed the manuscript. AP contributed to the development and testing of the application and reviewed the manuscript. SP contributed to the testing of the application and reviewed the manuscript. EC contributed to the testing of the application and reviewed the manuscript. RP contributed to the development and testing of the application and reviewed the manuscript. All authors read and approved the final manuscript.

## Pre-publication history

The pre-publication history for this paper can be accessed here:

http://www.biomedcentral.com/1472-6920/10/39/prepub

## Supplementary Material

Additional file 1 **Admin user survey questionnaire**. Instructions and survey questionnaire for admin users Click here for file

Additional file 2 **User survey questionnaire**. Instructions and survey questionnaire for users. Click here for file
