# Supplementary material for: WriteSim TCExam - An open source text simulation environment for training novice researchers in scientific writing
Source: BMC Med Educ. 2010 May 28;10:39. doi: 10.1186/1472-6920-10-39 (PMC2893514; doi:10.1186/1472-6920-10-39)
Supplement: Additional file 1 — Admin user survey questionnaire. Instructions and survey questionnaire for admin users [file 1472-6920-10-39-S1.DOCX]

| Additional File 1Admin user survey questionnaire  \| Instructions:  Kindly answer the following questions after the following steps: 1. View the video [tutorial](http://researchonresearch.blogspot.com/2008/08/tc-exam-textual-simulation-environment.html) 2. Access WriteSim TCExam application from the following links: Admin interface -<http://www.ceso.duke.edu/tcexam/admin/code/index.php> User interface - <http://www.ceso.duke.edu/tcexam/public/code/index.php>  Each of you will receive an email with your login details for both the interfaces.  3. Please do the following tasks \| \| \| --- \| --- \| \|  \|  \| \| **S.no.** \| **Task** \| \| 1 \| Admin interface \| \| A \| Log in with the details provided \| \| B \| In User section - add new user information, edit information and then delete the user \| \| C \| In User section - add new group information, edit information and then delete the group \| \| D \| In User section - view user selection and online subsections \| \| E \| In Topics section - add new topic information and then edit its information \| \| F \| In Topics section - add new question under the topic that you had created and then edit its information \| \| G \| In Topics section - add new answer and its keys under the question that you had created and then edit its information \| \| H \| In Topics section - view and navigate in the "list" sub section \| \| I \| In the Topics section delete the topic that you had created \| \| J \| In the Test section - add a new test and then edit its information \| \| K \| In the Test section - view the "evaluation," "results," "users," and "statistics" subsections \| \| M \| Log out from Admin interface \| \| 2 \| User Interface \| \| A \| Log in with the details provided \| \| B \| View the interface \| \| C \| Take the "Demo" test \| \| D \| Log out from User interface \| \|  \| \| \| 1. The speed of the application (WriteSim) is excellent.  Strongly disagree  Disagree  Neutral  Agree  Strongly agree \| \| \| 2. WriteSim is extremely easy to learn  Strongly disagree  Disagree  Neutral  Agree  Strongly agree \| \| \| 3. WriteSim is extremely easy to use  Strongly disagree  Disagree  Neutral  Agree  Strongly agree \| \| \| 4. WriteSim is extremely easy to use    Strongly disagree  Disagree  Neutral  Agree  Strongly agree \| \| \| 5. The navigation in WriteSim is highly intuitive  Strongly disagree  Disagree  Neutral  Agree  Strongly agree \| \| |  |
| --- | --- | --- | --- | --- | --- | --- | --- | --- | --- | --- | --- | --- | --- | --- | --- | --- | --- | --- | --- | --- | --- | --- | --- | --- | --- | --- | --- | --- | --- | --- | --- | --- | --- | --- | --- | --- | --- | --- | --- | --- | --- | --- | --- | --- | --- | --- | --- | --- | --- | --- | --- | --- | --- | --- | --- |
|  | |
|  | |
|  | |
| \| **The following statements relate to your skill with computers. This section will focus on evaluating "Beginning Level Computer Skills." Please use the following scale and, for each statement, circle the response that best describes your current belief.**    1. Strongly disagree   2. Disagree   3. Neither agree nor disagree   4. Agree   5. Strongly Agree \| \| --- \| \| a. “***I feel confident”*** Adding and deleting information from a data file  Strongly disagree  Disagree  Neither agree nor disagree  Agree  Strongly agree \| \| b. “***I feel confident”*** Escaping/exiting from the program/software  Strongly disagree  Disagree  Neither agree nor disagree  Agree  Strongly agree \| \| c. “***I feel confident”*** Copying an individual file  Strongly disagree  Disagree  Neither agree nor disagree  Agree  Strongly agree \| \| d. “***I feel confident”*** Copying a disk  Strongly disagree  Disagree  Neither agree nor disagree  Agree  Strongly agree \| \| e. “***I feel confident”*** Making selections from an onscreen menu  Strongly disagree  Disagree  Neither agree nor disagree  Agree  Strongly agree \| \| f. “***I feel confident”*** Moving the cursor around the monitor screen  Strongly disagree  Disagree  Neither agree nor disagree  Agree  Strongly agree \| \| g. “***I feel confident”*** Using a printer to make a hard copy of my work  Strongly disagree  Disagree  Neither agree nor disagree  Agree  Strongly agree \| \| h. “***I feel confident”*** Using the computer to write a letter or essay  Strongly disagree  Disagree  Neither agree nor disagree  Agree  Strongly agree \| \| i. “***I feel confident”*** Handling a floppy disk correctly  Strongly disagree  Disagree  Neither agree nor disagree  Agree  Strongly agree \| \| j. “***I feel confident”*** Entering and saving data (numbers or words) into a file  Strongly disagree  Disagree  Neither agree nor disagree  Agree  Strongly agree \| \| k. “***I feel confident”*** Getting the software up and running  Strongly disagree  Disagree  Neither agree nor disagree  Agree  Strongly agree \| | |
