# Supplementary material for: WriteSim TCExam - An open source text simulation environment for training novice researchers in scientific writing
Source: BMC Med Educ. 2010 May 28;10:39. doi: 10.1186/1472-6920-10-39 (PMC2893514; doi:10.1186/1472-6920-10-39)
Supplement: Additional file 2 — User survey questionnaire. Instructions and survey questionnaire for users. [file 1472-6920-10-39-S2.DOC]

**Additional file 2**

User survey questionnaire

| **General information** |
| --- |
| Gender    Male  Female |
| Education  Undergraduate  Graduate  Post graduate |
| Past peer reviewed publications   Never published  Published 1 scientific manuscript  Published more than 1 but less than 5   More than 5 publications |

| **The following statements relate to your self-efficacy with computers. This section will focus on evaluating "Beginning Level Computer Skills" Please use the following scale and, for each statement, circle the response that best describes your current belief.**    1. Strongly disagree   2. Disagree   3. Neither agree nor disagree   4. Agree   5. Strongly Agree |
| --- |
| a. “***I feel confident?”*** with Adding and deleting information from a data file  Strongly disagree  Disagree  Neither agree nor disagree  Agree  Strongly agree |
| b. “***I feel confident?”*** Escaping/exiting from the program/software  Strongly disagree  Disagree  Neither agree nor disagree  Agree  Strongly agree |
| c. “***I feel confident?”*** copying an individual file   Strongly disagree  Disagree  Neither agree nor disagree  Agree  Strongly agree |
| d. “***I feel confident?”*** copying a disk   Strongly disagree  Disagree  Neither agree nor disagree  Agree  Strongly agree |
| e. “***I feel confident?”*** Making selections from an onscreen menu   Strongly disagree  Disagree  Neither agree nor disagree  Agree  Strongly agree |
| f. “***I feel confident?”*** Moving the cursor around the monitor screen   Strongly disagree  Disagree  Neither agree nor disagree  Agree  Strongly agree |
| g. “***I feel confident?”*** Using a printer to make a “hardcopy” of my work   Strongly disagree  Disagree  Neither agree nor disagree  Agree  Strongly agree |
| h. “***I feel confident?”*** Using the computer to write a letter or essay   Strongly disagree  Disagree  Neither agree nor disagree  Agree  Strongly agree |
| i. “***I feel confident?”*** Handling a floppy disk correctly   Strongly disagree  Disagree  Neither agree nor disagree  Agree  Strongly agree |
| j. “***I feel confident?”*** Entering and saving data (numbers of words) into a file  Strongly disagree  Disagree  Neither agree nor disagree  Agree  Strongly agree |
| j. “***I feel confident?”*** Entering and saving data (numbers of words) into a file  Strongly disagree  Disagree  Neither agree nor disagree  Agree  Strongly agree |
| j. “***I feel confident?”*** Entering and saving data (numbers of words) into a file  Strongly disagree  Disagree  Neither agree nor disagree  Agree  Strongly agree |

| **WriteSim application** Kindly answer the questions after implementing the following steps: 1. Access WriteSim application from the link:  <http://www.ceso.duke.edu/tcexam/public/code/index.php>  Each of you will already have received an email with your login details. 2. Take the demo test 3. Based on your observations about the User interface and simulation material on scientific manuscripts provide answers to questions below |
| --- |
| 1. The speed of the application (WriteSim) is excellent.  Strongly disagree  Disagree  Neutral  Agree  Strongly agree |
| 2.  The navigation in WriteSim is highly intuitive  Strongly disagree  Disagree  Neutral  Agree  Strongly agree |
| 3. WriteSim is extremely easy to use  Strongly disagree  Disagree  Neutral  Agree  Strongly agree |
|  |

| **Related to simulation material** |
| --- |
| 1. Based on your short interaction with the simulation material,  do you think it helped you better understand the role of various subsections of a scientific manuscript for clinical research?   Strongly disagree  Disagree  Neutral  Agree  Strongly agree |
| 2. Based on your short interaction with the simulation environment, do you think it helped you better understand how specific scientific content fits into different subsections of a scientific manuscript?   Strongly disagree  Disagree  Neutral  Agree  Strongly agree |
| 3. Do you think the keys (feedback mechanism) in the simulation tests are highly beneficial?     Strongly disagree  Disagree  Neutral  Agree  Strongly agree |
| 4. The simulation material in Writesim is useful for learning manuscript writing related to clinical research.  Strongly disagree  Disagree  Neutral  Agree  Strongly agree |
| 5. Based on your short interaction with the simulation environment, would you look forward to use this application in the future?  Yes  No |
| Please provide suggestions/comments related to the simulation material (if any)? |
